# 1106. Comparison of Diagnostic Power of Conventional Culture and Automated Blood Culture System with Pleural Fluid

**DOI:** 10.1093/ofid/ofad500.079

**Published:** 2023-11-27

**Authors:** Young Ho Lee, Si-Ho Kim, Jinyoung Yang, Jae-Hoon Ko, Sun Young Cho, Heejae Huh, Nam Yong Lee, Cheol-In Kang, Doo Ryeon Chung, Kyong Ran Peck, Kyungmin Huh

**Affiliations:** Samsung Medical Center, Seoul, Seoul-t'ukpyolsi, Republic of Korea; Samsung Medical Center, Seoul, Seoul-t'ukpyolsi, Republic of Korea; Samsung Medical Center, Seoul, Seoul-t'ukpyolsi, Republic of Korea; Samsung Medical Center, Seoul, Seoul-t'ukpyolsi, Republic of Korea; Samsung Medical Center, Seoul, Korea, Seoul, Seoul-t'ukpyolsi, Republic of Korea; Department of Laboratory Medicine and Genetics, Samsung Medical Center, Sungkyunkwan University School of Medicine, Seoul, Seoul-t'ukpyolsi, Republic of Korea; Samsung Medical Center, Seoul, Seoul-t'ukpyolsi, Republic of Korea; Samsung Medical Center, Seoul, Seoul-t'ukpyolsi, Republic of Korea; samsung medical center, Seoul, Seoul-t'ukpyolsi, Republic of Korea; Samsung Medical Center, Seoul, Seoul-t'ukpyolsi, Republic of Korea; samsung medical center, Seoul, Seoul-t'ukpyolsi, Republic of Korea

## Abstract

**Background:**

Microbiological diagnosis of empyema is often hindered by low sensitivity of conventional culture. Automated blood culture system has been reported to enhance diagnostic power for culture of non-blood specimens. The purpose of this study is to compare the diagnostic power of conventional bacterial culture and automated blood culture system (ABCS) in patients who underwent thoracentesis or percutaneous drainage.

**Methods:**

Non-duplicate patients whose pleural fluid was tested using both conventional culture and ABCS (BACT/ALERT 3D and VIRTUO, bioMerieux) from 1 Jan 2001 to 31 Dec 2021 were included. Cases in which coagulase-negative staphylococci were only positive isolates were excluded. Culture results, demographic information, and laboratory test results were obtained from the clinical data warehouse of Samsung Medical Center. The results from conventional cultures were compared against those from ABCS.

**Results:**

A total of 9,020 patients who met the study criteria were identified. Among them, 819 patients had positive results other than coagulase-negative staphylococci from ABCS. Conventional culture was also positive in 360 (44.0%) patients, while 459 (56.0%) patients had isolates only from ABCS.

Pleural fluid from female and the patients with no pre-existing pleural drainage were significantly more likely to be culture positive by ABCS only (Table 1 and 2). In addition, pleural fluid LDH and serum CRP were significantly lower and pleural fluid glucose was significantly high in the patients with positive results from ABCS only. Among organisms isolated from both conventional culture and ABCS, the most frequently identified species was *Staphylococcus aureus*, followed by *Klebsiella* spp., and viridans group streptococci (Figure 1). Viridans group streptococci, *Candida* spp., and *S. aureus* were the most common species isolated from ABCS only (Figure 1).

**Figure:**
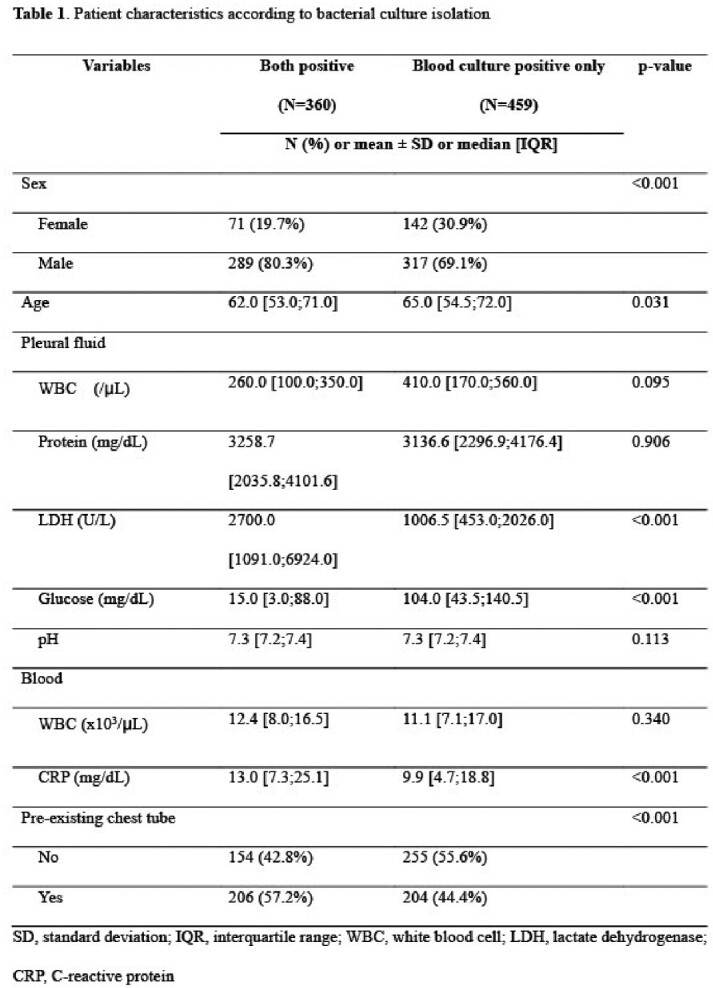

**Figure:**
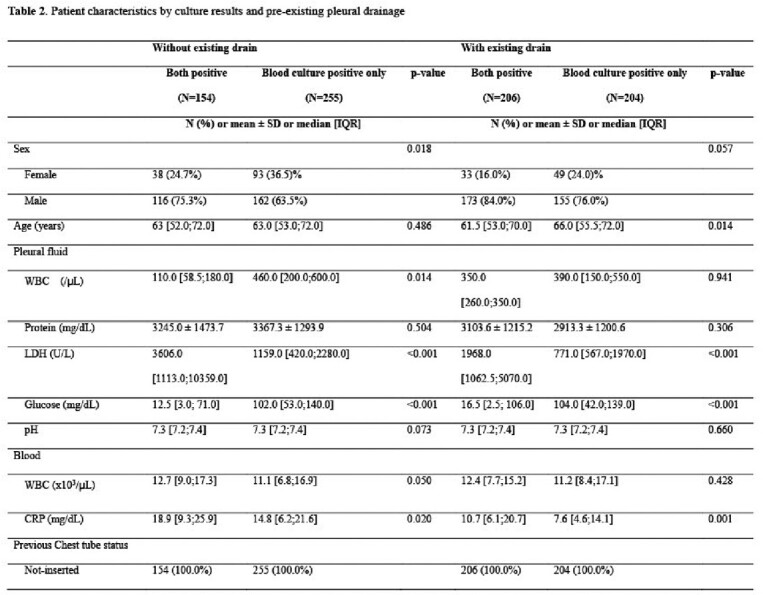

**Figure:**
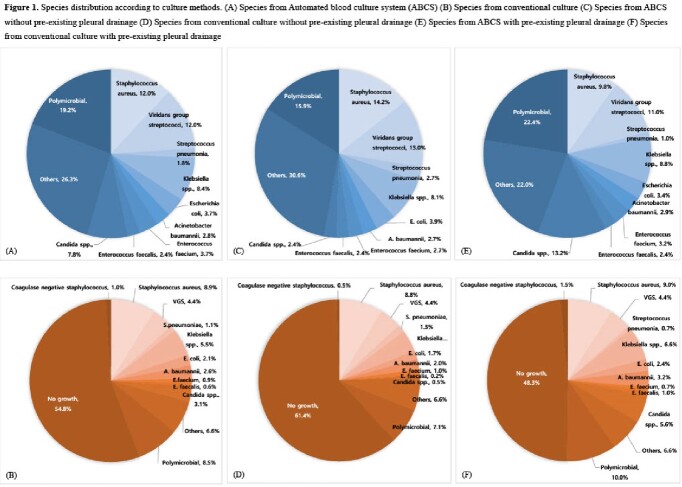

**Conclusion:**

More than a half of the patients whose pleural fluid culture were positive using ABCS had a negative conventional culture. Viridans group streptococci, *Candida* spp., and *S. aureus* were common species causing the discrepancy. Our results suggest that ABCS may enhance microbiologic diagnosis of empyema.

**Disclosures:**

**All Authors**: No reported disclosures

